# Molecular characteristics of the VP1 region of enterovirus 71 strains in China

**DOI:** 10.1186/s13099-020-00377-2

**Published:** 2020-08-14

**Authors:** Haiyan Sun, Min Gao, Dawei Cui

**Affiliations:** 1 Department of Clinical Laboratory, Shaoxing Second Hospital, Shaoxing, 312000 Zhejiang China; 2 Department of Laboratory Medicine, Huzhou Central Hospital, Huzhou, 313003 Zhejiang China; 3 Department of Blood Transfusion, The First Affiliated Hospital, Zhejiang University School of Medicine, Hangzhou, 310003 Zhejiang China; 4 Key Laboratory of Clinical In Vitro Diagnostic Techniques of Zhejiang Province, Hangzhou, China

**Keywords:** Molecular characteristics, Enterovirus 71, VP1, Mutation, Genotype

## Abstract

**Background:**

Enterovirus 71 (EV71) is the most commonly implicated causative agent of severe outbreaks of paediatric hand, foot, and mouth disease (HFMD).VP1 protein, a capsid protein of EV71, is responsible for the genotype of the virus and is essential for vaccine development and effectiveness. However, the genotypes of EV71 isolates in China are still not completely clear.

**Methods:**

The VP1 gene sequences of 3712 EV71 virus strains from China, excluding repetitive sequences and 30 known EV71 genotypes as reference strains, between 1986 and 2019 were obtained from GenBank. Phylogenetic tree, amino acid homology, genetic variation and genotype analyses of the EV71VP1 protein were performed with MEGA 6.0 software.

**Results:**

The amino acid identity was found to be 88.33%–100% among the 3712 EV71 strains, 93.47%–100% compared with vaccine strain H07, and 93.04%–100% compared with vaccine strains FY7VP5 or FY-23 K-B. Since 2000, the prevalent strains of EV71 were mainly of the C4 genotype. Among these, the C4a subgenotype was predominant, followed by the C4b subgenotype; other subgenotypes appeared sporadically between 2005 and 2018 in mainland China. The B4 genotype was the main genotype in Taiwan, and the epidemic strains were constantly changing. Some amino acid variations in VP1 of EV71 occurred with high frequencies, including A289T (20.99%), H22Q (16.49%), A293S (15.95%), S283T (15.11%), V249I (7.76%), N31D (7.25%), and E98K (6.65%).

**Conclusion:**

The C4 genotype of EV71 in China matches the vaccine and should effectively control EV71. However, the efficacy of the vaccine is partially affected by the continuous change in epidemic strains in Taiwan. These results suggest that the genetic characteristics of the EV71-VP1 region should be continuously monitored, which is critical for epidemic control and vaccine design to prevent EV71 infection in children.

## Introduction

Enterovirus 71 (EV71) is a common pathogen of hand, foot, and mouth disease (HFMD) in children, and it is also the most important risk factor for severe cases and deaths [1–4].Some children with HFMD can have severe neurological effects, such as aseptic meningitis, encephalitis and acute delayed paralysis, develop serious brainstem encephalitis, neurogenic pulmonary oedema and even die [5, 6]. Children with severe neurological diseases who survive often have irreversible sequelae, seriously threatening their health [7, 8].

EV71 was first discovered in 1969; the virus was distributed mainly in the Americas, Europe and other countries in a sporadic form, with outbreaks in some European countries in the 1970s and 1980s [9, 10]. After 1997, EV71 began to emerge and spread in Asia, and the Asia–Pacific region is the most prevalent area for EV71. Indeed, there are reports of EV71 outbreaks in China, Singapore, and Malaysia, among others [11, 12]. Diseases caused by EV71 infection have been widely prevalent in China since 2007 [13]. For example, HFMD pandemics in Linyi city, Shandong Province, and Guangdong, Anhui Province, in 2008 resulted in tens of thousands of childhood infections and death among dozens of children [14, 15].

EV71 belongs to the Enterovirus genus of the RNA virus family. EV71 can be clustered into three genotypes according to nucleotide differences in the VP1 region, including genotype A (BrCr) with only one member, B and C. In contrast, genotypes B and C are divided into five subgenotypes, B1–B5 and C1–C5, respectively, and genotype C4 is further subdivided into C4a and C4b [16–18]. In China, genotypeC4 is the main epidemic strain; C4b was the predominant epidemic genotype from 1998 to 2004 and the C4a subgenotype after 2004 in mainland China, though Taiwan strains continue to circulate, including C2, B4 and B5 genotypes [19–25]. Phase III clinical trials of vaccines from three companies in China have been completed, and the genotypes of their vaccine strains are all C4a subgenotypes [26]. The Vaccine Research and Development Center of National Institutes of Health in Taiwan has also developed an FI-EV71 vaccine based on the B4 subtype (EV71vac), which can cause a robust cross-neutralizing antibody reaction against different EV71 gene subtypes, such as B4, B1, B5 and C4a [26, 27].

Nonetheless, there are many reports on recombination between different genotypes of EV71 [28–31], suggesting that EV71 has high variability and recombination ability, which may lead to the production of new pathogenic strains. Therefore, genome monitoring of EV71 epidemic strains is of great significance for the prevention and control of EV71 epidemics and can guide the application of the EV71 vaccine to a certain extent. In this study, the VP1 sequences of all EV71 viruses registered in GenBank in China from 1996 to 2019 were collected, and the molecular characteristics of genes were analysed using bioinformatics software to provide a scientific basis for the prevention and control of HFMD epidemics.

## Methods

### Acquisition of the EV71VP1 gene sequence

**Fig. 1:**
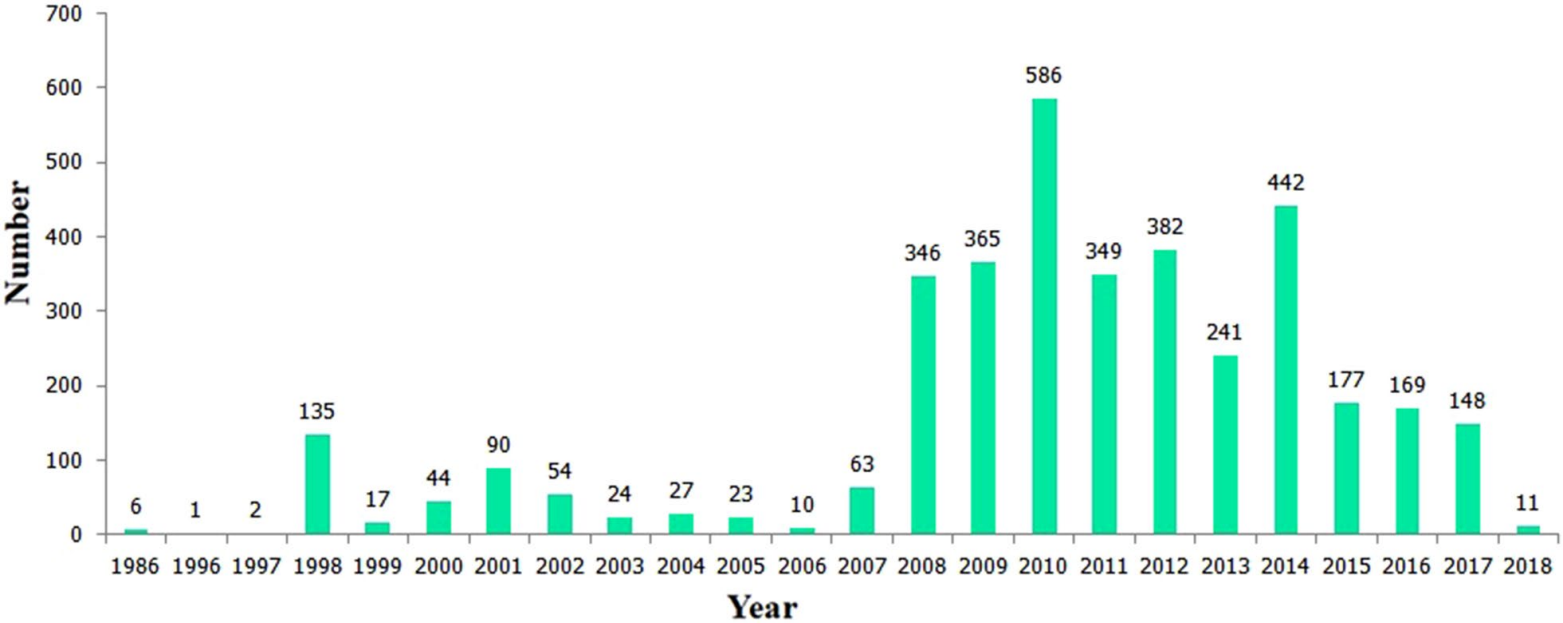
The number of EV71 strains isolated in different years in China. The 3712 EV71 strains with complete VP1 gene sequences collected in China were obtained from the GenBank public database at the National Center for Biotechnology Information (NCBI) PubMed website (http://www.ncbi.nlm.nih.gov/genbank/) between 1986 and December 31, 2019. However, no EV71 strains were available for 2019

**Fig. 2:**
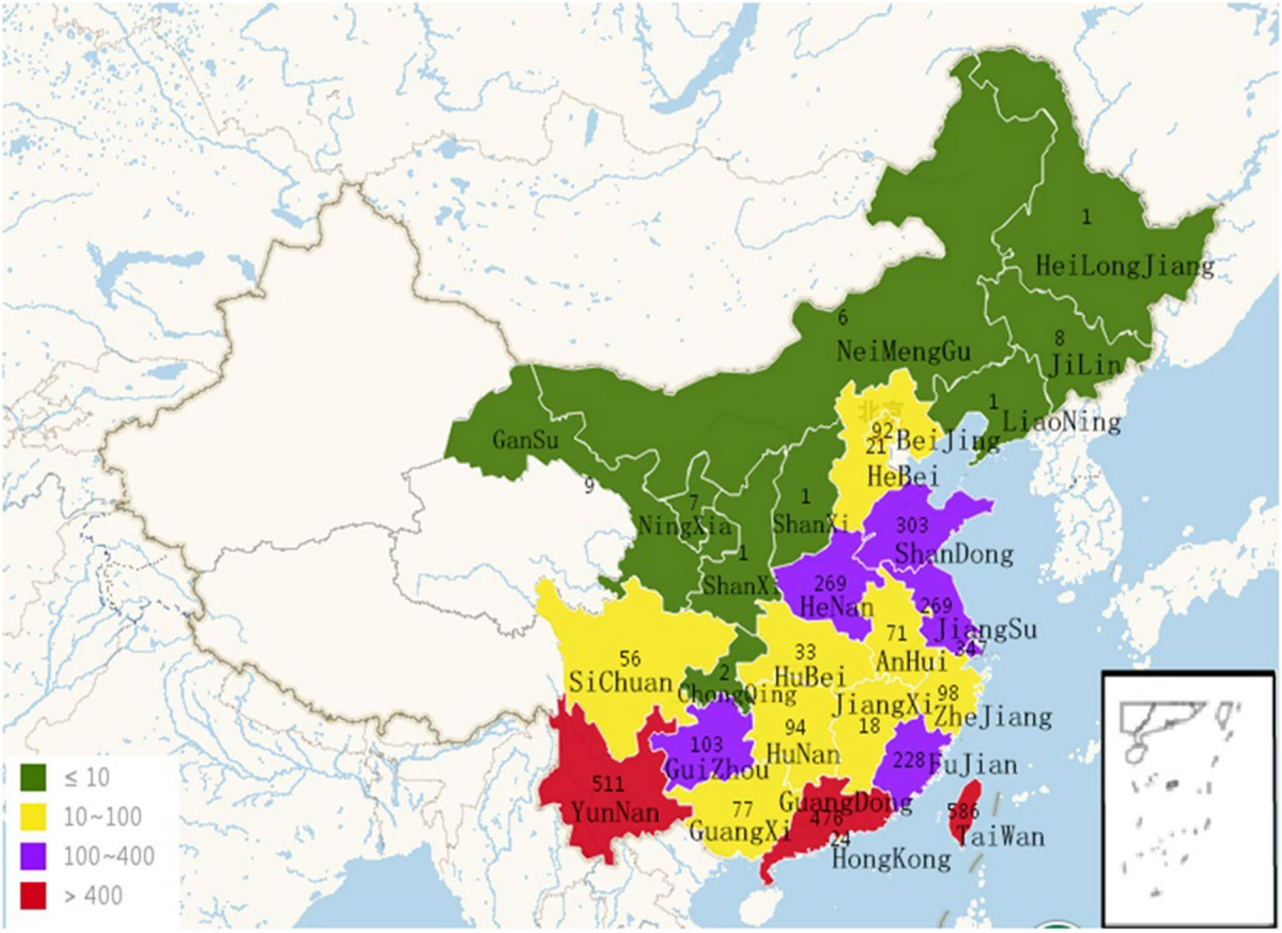
The number of EV71 strains isolated in different provinces of China. A total of 3712 EV71 strains were collected in China, including different provinces of mainland China (3102), Hong Kong (24), Macao (0) and Taiwan (586), between 1986 and December 31, 2019

The complete 891-bp VP1 gene sequence of EV71 strains isolated from children with HFMD from 1986 to December 31, 2019, in China with known collection dates and isolation regions, including mainland China, Hong Kong, Macao and Taiwan, was obtained from the GenBank public database at the National Center for Biotechnology Information (NCBI) PubMed website (http://www.ncbi.nlm.nih.gov/genbank/). A total of 8340 EV71 strains from China were collected from GenBank, consisting of 6572 strains from mainland China, 26 from Hong Kong, none from Macao, and 1742 from Taiwan. If EV71 strains were isolated the same year and from the same region and had 100% nucleotide homology, only one strain was included. Therefore, a total of 3470 strains from mainland China, 2 from Hong Kong, and 1156 from Taiwan were removed. Ultimately, 3712 strains, consisting of 3102 from mainland China, 24 from Hong Kong, and 586 from Taiwan, were retained for the study. The time and geographical distribution of all EV71 isolates are shown in Figs. 1 and 2. The nucleotide sequence of the complete VP1 gene of all virus strains is 891 bp, and Chinese vaccine strains (H07, FY7VP5, and FY-23 K-B) and 27 known genotypes of EV71 strains were considered reference sequences, as presented in Table 1.

**Table 1:** Data of reference strains of EV71 genotypes

No.	GenBank No.	Year of isolation	Place of isolation	Genotype
1	HQ328793 (H07)	2008	China	C4a
2	JX025561 (FY7VP5)	2008	China	C4a
3	EU812515 (FY-23 K-B)	2008	China	C4a
4	HQ828086	2010	China	C4a
5	EU753365	2007	China	C4a
6	KU936124	2014	China	C4a
7	KU936125	2014	China	C4a
8	KU936130	2014	China	C4a
9	JQ742002	2001	China	C4b
10	JQ742001	2001	China	C4b
11	AF376081	1998	Malaysia	C3
12	AY207625	2000	Malaysia	C3
13	AF304457	1998	Taiwan	C2
14	AF376110	1999	Australia	C2
15	AY125969	2002	Korea	C1
16	AY125976	2002	Korea	C1
17	JN874558	2007	Taiwan	C5
18	KU888174	2008	Vietnam	C5
19	U22521	1970	USA	A
20	GU434678	2009	China	A
21	AF135886	1974	Australia	B1
22	AB059814	1975	Bulgaria	B1
23	AF009540	1988	USA	B2
24	AF009534	1987	USA	B2
25	AF376119	1998	Singapore	B3
26	AF376073	1997	Malaysia	B3
27	AJ586873	1997	Malaysia	B4
28	AF376084	2000	Malaysia	B4
29	AB177815	2003	Japan	B5
30	AB177816	2003	Japan	B5

### Construction of an EV71VP1 phylogenetic tree and gene sequence analysis

The complete VP1 sequence of EV71 strains was compared by Molecular Evolutionary Genetics Analysis (MEGA) version 6.0 [32]. In brief, a phylogenetic tree of the EV71VP1 gene was constructed by the adjacency method (neighbour-joining, N-J) with 1000 bootstrap replications. Homology and variation of EV71 strain VP1 gene sequences were analysed by MEGA 6.0.

## Results

### Phylogeny and homology analysis of the EV71 VP1 region

**Fig. 3:**
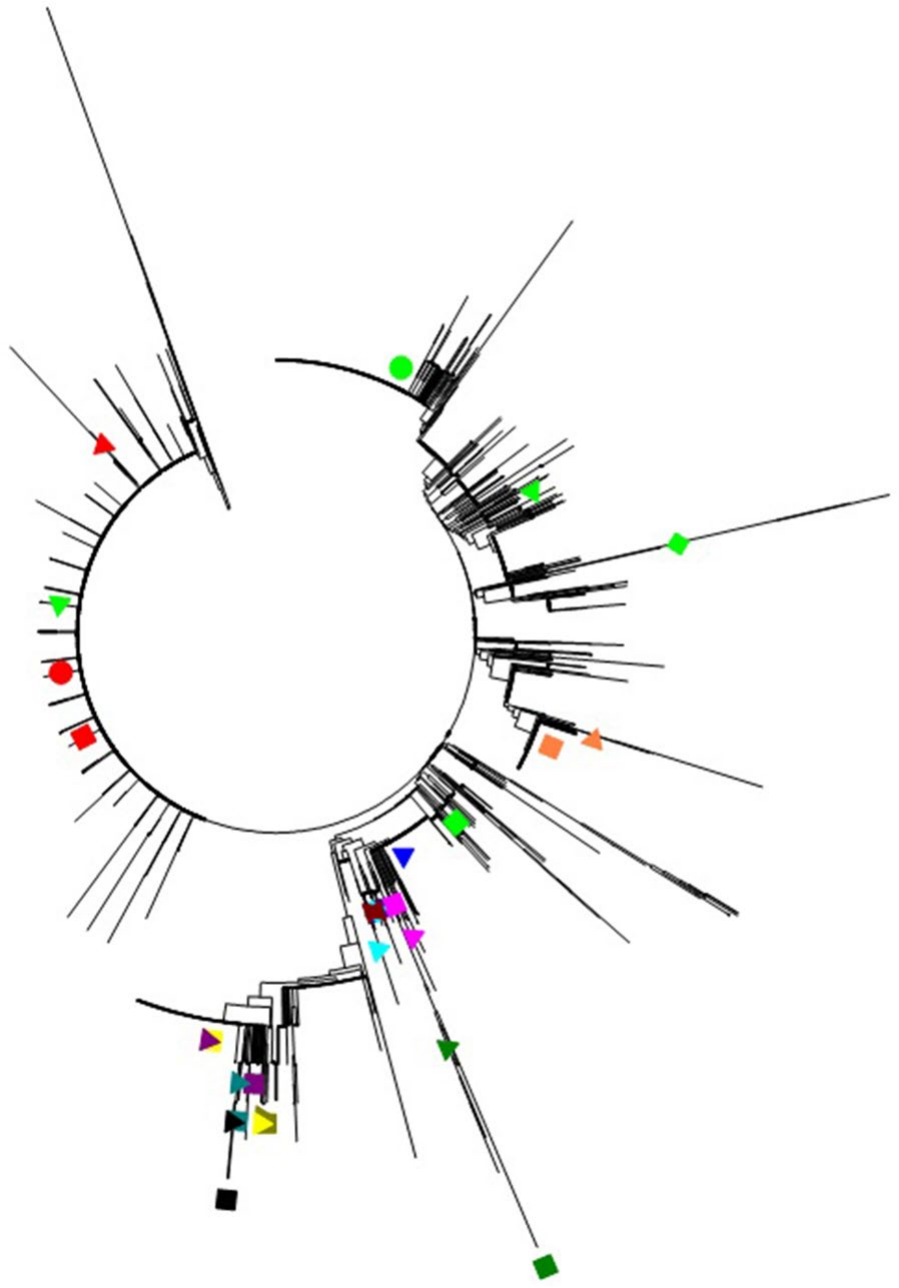
Phylogenetic tree of the entire VP1 gene of EV71. The 30 reference strains are indicated in different coloured shapes, as shown below:  :HQ328793(H07)  :JX025561(FY7VP5)  :EU812515(FY-23 K-B)  :HQ828086(C4a)  :EU753365(C4a)  :KU936124(C4a)  :KU936125(C4a)  :KU936130(C4a)  :JQ742002(C4b)  :JQ742001(C4b)  :AY207625(C3)  :AF376081(C3)  :AF304457(C2)  :AF376110 (C2)  :AY125969(C1)  :AY125976 (C1)  :JN874558 (C5)  :KU888174(C5)  :U22521(A)  :GU434678(A)  :AB059814(B1)  :AF135886 (B1)  :AF009534(B2)  :AF009540(B2)  :AF376119(B3)  :AF376073(B3)  :AF376084(B4)  :AJ586873 (B4)  :AB177815 (B5)  :AB177816 (B5)

A phylogenetic tree of the VP1 amino acid sequence was constructed with 3712 EV71 isolates from China and 30 reference strains (Fig. 3). Amino acid identity among the 3712 EV71 strains is 88.33%–100%, 93.47%–100% compared with vaccine strain H07 and 93.04%–100% compared with vaccine strains FY7VP5 or FY-23 K-B. Among the EV71 strains, the C4 genotype accounted for most of the EV71 strains, and the C4a subgenotype was the most common. Moreover, B4, C4b and C2 were found to be important genotypes of EV71; other subgenotypes appeared sporadically.

### Genotypic distribution of EV71 strains in different years in China

**Fig. 4:**
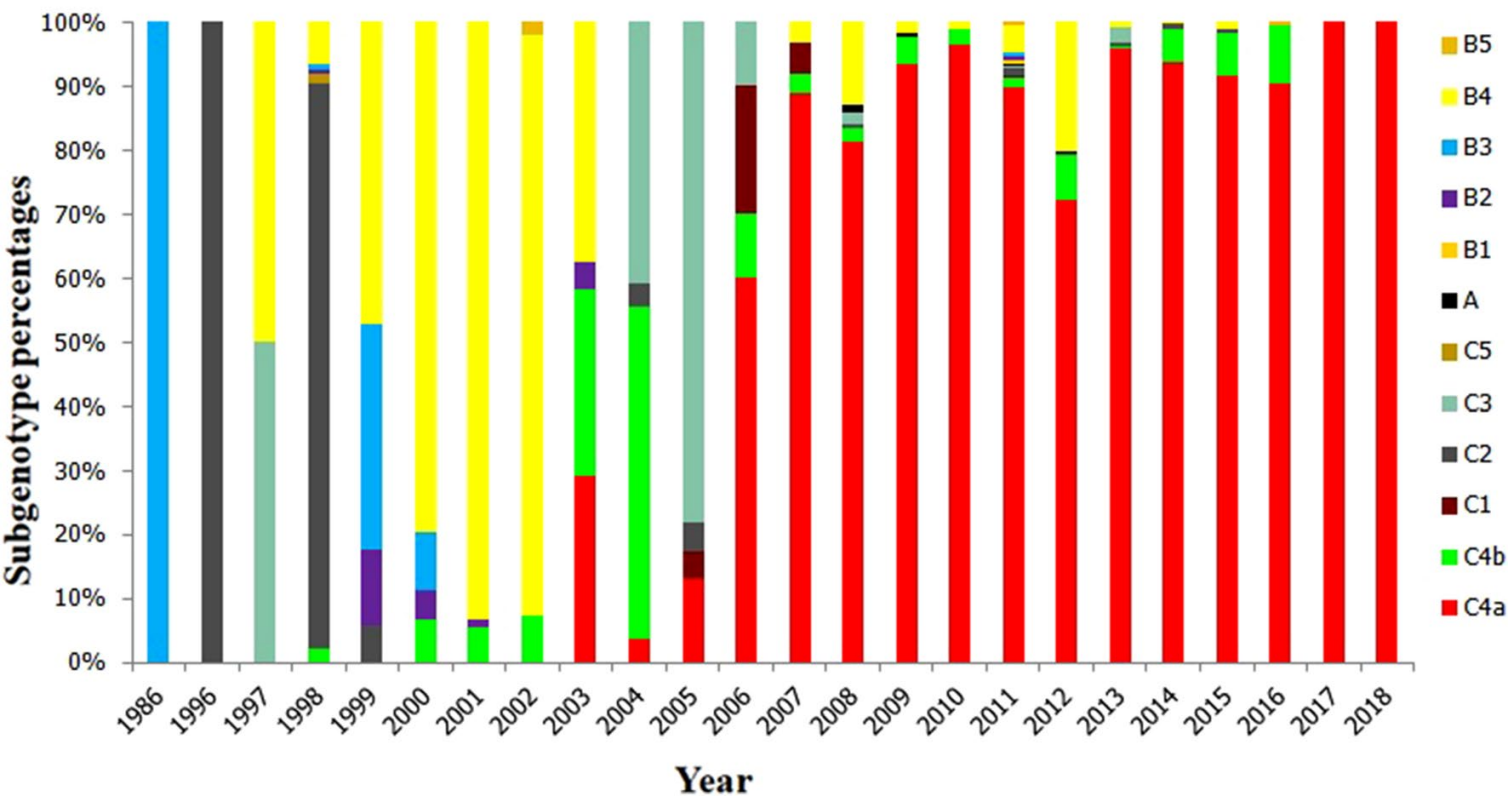
Subgenotype percentages of EV71 for every year in China. In this study, 12 genotypes were detected among 3712 strains. The B4 genotype was the predominant strain between 1999 and 2003 and C3 genotypes from 2004 to 2005; the C4a subgenotype was the most common epidemic strain from 2006 to 2018

Among the 3712 strains, the first 6 strains isolated from China in 1986were all of the B3 genotype, after which different genotypes/subgenotypes were detected in China. From 1986 to 2007, there was a small epidemic peak of C2-genotype EV71 in 1998. Between 1999 and 2003, the B4 genotype was the predominant strain of EV71. Most EV71 strains between 2004 and 2005 were of the C3 genotype, and from 2006 to 2018, the C4a subgenotype was the most common epidemic strain. B4 and C4b genotypes also accounted for a certain proportion (Table 2 and Fig. 4).

**Table 2:** Number of EV71 genotypes for every year in China

Year	Genotypes of EV71												Number
	C4a	C4b	C1	C2	C3	C5	A	B1	B2	B3	B4	B5	
1986										6			6
1996				1									1
1997					1						1		2
1998		3		119		2			1	1	9		135
1999				1					2	6	8		17
2000		3							2	4	35		44
2001		5							1		84		90
2002		4									49	1	54
2003	7	7							1		9		24
2004	1	14		1	11								27
2005	3		1	1	18								23
2006	6	1	2		1								10
2007	56	2	3								2		63
2008	281	8		2	6		4				45		346
2009	341	16					2				6		365
2010	565	15									6		586
2011	313	6	1	4	1		1	2	2	3	14	2	349
2012	276	27		1			1				77		382
2013	231	1		1	6						2		241
2014	415	22		4							1		442
2015	162	12		1							2		177
2016	153	15										1	169
2017	148												148
2018	11												11

**Fig. 5:**
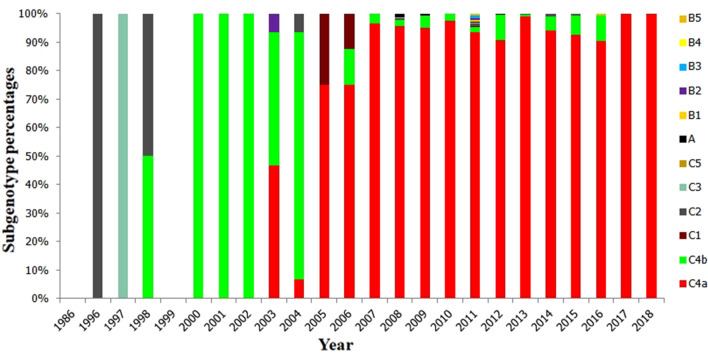
The genotypic distribution of EV71 every year in mainland China. The C4a subgenotype was absolutely the predominant strain of EV71 from 2005 to 2018, followed by theC4b subgenotype; other subgenotypes appeared sporadically in mainland China

Next, the EV71 strains isolated from China, including mainland China, Hong Kong, and Taiwan, were further analysed to explore their genotypic distribution in different years. The results indicated that from 2005 to 2018, the C4a subgenotype was absolutely the predominant strain of EV71, followed by the C4b subgenotype; other subgenotypes appeared sporadically in mainland China. Moreover, among the 24 strains isolated from Hong Kong, there were 10 C4a-subgenotype strains (1 strain in 2008, 8 strains in 2010, and 1 strain in 2012) and 14 C4b-subgenotype strains (1 strain in 2008 and 13 strains in 2010). The results for mainland China are shown in Table 3 and Fig. 5.

**Table 3:** Number of EV71 genotypes for every year in mainland China

Year	Gene subtype of EV71												Number
	C4a	C4b	C1	C2	C3	C5	A	B1	B2	B3	B4	B5	
1986													0
1996				1									1
1997					1								1
1998		1		1									2
1999													0
2000		3											3
2001		4											4
2002		4											4
2003	7	7							1				15
2004	1	13		1									15
2005	3		1										4
2006	6	1	1										8
2007	56	2											58
2008	280	6		2	1		4						293
2009	341	16					2						359
2010	551	15											566
2011	302	6	1	3	1		1	2	2	3		2	323
2012	273	27					1						301
2013	231	1		1									233
2014	415	22		4									441
2015	159	12		1									172
2016	148	15										1	164
2017	148												148
2018	11												11

**Fig. 6:**
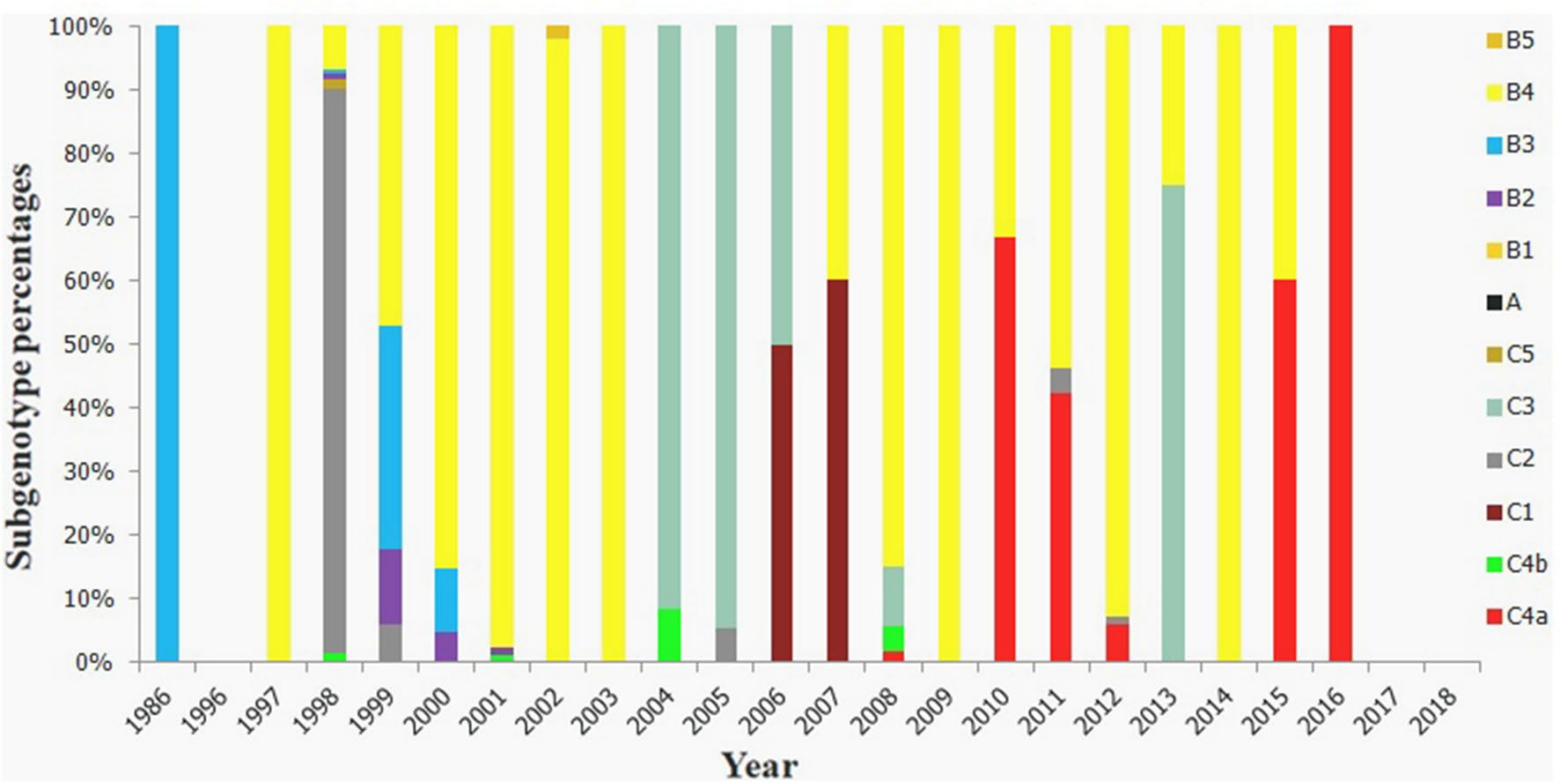
The subgenotype percentages of EV71 for every year in Taiwan. Epidemic strains of EV71 in Taiwan in recent years are constantly changing. The B3 genotype first emerged in 1986. From 1999 to 2003, 2008 to 2009, and 2011 to 2012, the B4 genotype was the predominant strain. Between 2010 and 2011, C4a was the main genotype

However, the epidemic strains of EV71 in Taiwan from 1986 to 2018 are quite different from those in mainland China. Overall, epidemic strains are not predominantly represented by a single subgenotype but are constantly changing. The first 6 strains isolated in Taiwan in 1986 were all of the B3 genotype. In 1998, C2 was the predominant EV71 genotype in Taiwan. From 1999 to 2003, 2008 to 2009, and 2011 to 2012, the epidemic strains in Taiwan evolved mainly into B4-genotype EV71 strains. Additionally, C4a was an important genotype between 2010 and 2011 (Table 4 and Fig. 6).

**Table 4:** Number of EV71 genotypes for every year in Taiwan, China

Year	Gene subtype of EV71												Number
	C4a	C4b	C1	C2	C3	C5	A	B1	B2	B3	B4	B5	
1986										6			6
1996													0
1997											1		1
1998		2		118		2			1	1	9		133
1999				1					2	6	8		17
2000									2	4	35		41
2001		1							1		84		86
2002											49	1	50
2003											9		9
2004		1			11								12
2005				1	18								19
2006			1		1								2
2007			3								2		5
2008	1	2			5						45		53
2009											6		6
2010	12										6		18
2011	11			1							14		26
2012	5			1							77		83
2013					6						2		8
2014											1		1
2015	3										2		5
2016	5												5
2017													0
2018													0

### Analysis of amino acid variation of EV71VP1

Analysis of common amino acid variation sites in the EV71VP1 region showed that of the 3712 strains, residue 289 was the major common site, with a total variation rate of 23.55%; A289T was the most common variant, accounting for 20.99%. Moreover, 10.24% of strains from mainland China, including Hong Kong, exhibited this variation; 10.75% of strains from Taiwan also exhibited this variation. There was a total mutation rate of 21.85% at amino acid position 22 in the VP1 region. The frequency of the H22Q variation was detected to be 16.49%, of which mainland China, including Hong Kong, exhibited 5.50%; Taiwan exhibited 10.99%. Furthermore, 23% of the EV71 strains harboured an H22R mutation. The mutation rates of A293S, S283T, V249I, N31D and E98K were 15.95%, 15.11%, 7.76%, 7.76% and 6.65%, respectively. Among the common sites of amino acid variation in the EV71VP1 region, the most common was residue 293, with five types of amino acid variation, among which the A293S mutation was the most common (Table 5).

**Table 5:** Analysis of common amino acid variation sites in VP1 of EV71

Common amino acid	22H		31 N		98E		145E		249 V	
Mutant amino acids	Q	16.49%	D	7.25%	K	6.65%	G	3.13%	I	7.76%
	R	5.23%	S	0.13%	G	0.08%	Q	3.50%	A	0.05%
	N	0.13%	G	0.03%	R	0.03%	A	0.30%	F	0.03%
			R	0.03%			L	0.05%		
	Total	21.85%	Total	7.44%	Total	6.76%	Total	6.98%	Total	7.84%
Common amino acid	262I		282 N		283S		289A		293A	
Mutant amino acids	V	2.77%	S	1.78%	T	15.11%	T	20.99%	S	15.95%
	Q	0.03%	D	0.32%	A	0.08%	V	2.32%	V	0.13%
			K	0.08%	F	0.05%	D	0.16%	G	0.08%
			T	0.05%			I	0.08%	L	0.03%
									P	0.03%
	Total	2.80%	Total	2.24%	Total	15.25%	Total	23.55%	Total	16.22%

H, histidine; Q, glutamine; R, arginine; N, asparagine; D, aspartic acid; S, serine; G, glycine; E, glutamic acid; K, lysine; A, alanine; L, leucine; V, valine; I, isoleucine; F, phenylalanine; T, threonine; P, proline

## Discussion

It has been reported that since 2004, the major EV71 strains in mainland China basically belong to the C4 genotype [18, 33]. In this study, it was found that the epidemic strains of EV71 in mainland China have been dominated by the C4 genotype since 2000, mainly the C4b subgenotype from 2000 to 2004, and the proportion of the C4a subgenotype has increased significantly since 2005.From 2005 to 2018, the C4a subtype was dominant; the C4b subtype was the second most common, and other subtypes appeared sporadically; this pattern was similar to previous reports [20, 21, 30, 33]. From 2004 to 2018, most of the EV71 isolates in mainland China were genotype C, and most of them were subtype C4 (especially subtype C4a); other genotypes occurred sporadically (9 strains of genotype B in 2011, 4 strains of genotype A in 2008, 2 strains of genotype A in 2009, 1 strain of genotype An in 2011 and 1 strain of genotype A in 2012). The immune effectiveness of inactivated vaccines mostly depends on the antigenic correlation between epidemic strains and vaccine strains, which are often best for preventing infection of the same subtype virus but are inferior against different subtypes [30]. Recent studies have also shown that the EV71 vaccine (especially in children who receive 2 doses) can effectively prevent and control childhood EV71-associated HFMD but has no protective effect against coxsackievirus (CV) A6 (CVA-6) or CVA16, and there is no explanation for the effectiveness of other subtypes of EV71 (excluding C4a subtypes) [34]. These studies show that vaccine research and development for EV71 combined with CVA6 and CVA16 and other multivalent vaccines might better prevent EV71 infection.

Interestingly, this study found that the EV71 epidemic strains in Taiwan were mainly of the B4 genotype, which was different from those in mainland China; EV71 epidemic strains are also constantly changing, which is consistent with a previous report [26]. According to a human phase 1 clinical trial on adults in 2010, the FI-EV71 vaccine (EV71vac) based on the B4 genotype from Taiwan is safe and induces a high titre of neutralizing antibodies against EV71; it was also highly effective against B1, B5, and C4a strains. However, the titres of neutralizing antibodies against C4b and CVA16 were low in 20% of volunteers, and virus-neutralizing antibodies against the C2 genotype were not detected in 90% of vaccine recipients [26, 27]. These studies indicate that it is necessary to strengthen the monitoring of EV71 genotypes; new multivalent and effective vaccines that can cover local strains should be designed and applied according to the genotypes of the local predominant EV71 epidemic strains to ensure that the vaccine is more accurate in controlling HFMD epidemics.

Some studies have shown that the H22Q mutation in the VP1 protein of EV71 can lead to a decrease in the adsorption capacity of the C4 genotype to host cells [35–37]. The amino acid at position 22 of 78.15% of the 3712 strains isolated in China is H (histidine), which suggests that most of the viruses have strong adsorption capacity to host cells. Furthermore, H22Q was detected in 10.99% of all EV71 strains in Taiwan, significantly more prevalent than that in mainland China and Hong Kong (5.50%), suggesting that the adsorption capacity of some strains in Taiwan to host cells is weak in comparison with that of strains in mainland China.

Studies have shown that the A289T EV71VP1 variant is closely related to the occurrence of severe HFMD and that the neurological symptoms caused by EV71 infection are significantly increased when the amino acid at position 289 of VP1 is A (alanine); in contrast, there is low neurotoxicity when the amino acid is T (threonine) [36, 38]. In this study, 76.45% (2838 strains) of the virus strains were found to contain an A (alanine), suggesting that most of these EV71 viruses have high neurotoxicity. Moreover, 10.24% (380 strains) of the strains in mainland China (including Hong Kong) and 10.75% (399 strains) of those in Taiwan contain a T (threonine), suggesting low neurotoxicity. It remains to be further studied whether new mutations such as A289V (valine)/D (aspartic acid)/I (isoleucine) mutations will cause the emergence of severe HFMD.

EV71 can infect human lymphocytes by binding to its receptor molecule P-selectin glycoprotein ligand-1 (PSGL-1). When E (glutamic acid) at position 145 in VP1 is mutated to G (glycine) or Q (glutamine), the virus binds PSGL-1 more readily, whereas its PSGL-1-binding ability is weakened or lost when E is present [39]. In this study, the amino acid at position 145 in most strains was found to be E, with an E145G/Q mutation rate of 6.63%, suggesting that the emergence of this mutation may result in a virus that is more likely to infect human lymphocytes.

It has been reported that the E98K mutation may increase the hydrophobicity of VP1, making it easy for large compounds to enter and interfere with receptor binding, suggesting that E98K mutant viruses are sensitive to larger compounds [40]. Other studies have shown that E145G and N31D mutations are associated with increased virulence of EV71 and may increase the risk of neurological complications but that I262V mutations reduce the risk of neurological complications [41–43]. In this study, the E98K, E145G, N31D and I262V mutation rates were 6.65%, 3.13%, 7.25% and 2.77%, respectively. These findings indicate that these mutations may play an important role in the pathogenicity of mild and severe EV71-associated HFMD.

Humans are the only natural host and source of EV71. Indeed, EV71 cannot infect rodents, which is due mainly to the incompatibility between the virus and rodent cells, and the different expression of its scavenger receptor in humans and rodents [44–46]. However, some studies have found that simultaneous substitution of K98E, E145A and L169F in VP1 of EV71 can result in infection in mice [44]. Our study showed that among 3712 strains, the mutation frequencies of K98E, E145A and L169F were 93.24%, 0.30% and 0.03%, respectively; however, no strain with all three mutations was found. These findings indicate that humans are still the only host of EV71 in China; nevertheless, the existence of individual mutations does not rule out the emergence over time of strains that can infect other mammals. Therefore, it is important to closely monitor mutation of the key sites of the EV71VP1 protein.

EV71 is the most important pathogen causing severe HFMD in children, which can lead to irreversible sequelae or death, and it is a serious threat to their health [4, 7]. At present, there is no specific treatment for EV71 infection. The development and marketing of an inactivated EV71 vaccine in China is crucial for the prevention of HFMD caused by EV71 infection [26, 47–49]. Phase III clinical trials of the EV71 inactivated vaccine approved in China in 2015 have shown protective effectiveness against EV71-associated HFMD of more than 90% [47, 50–52]. However, according to molecular epidemiological studies of EV71, EV71 gene mutations occur frequently, leading to genetic diversity [28–31, 53]. These studies suggest that there is still a need for strengthening surveillance of EV71 genotypes and the development of new EV71 vaccines.

This study had a retrospective design, and there are some limitations. First, the EV71VP1 gene sequences from China analysed in this study were downloaded from the GenBank database but were not tested by us. Due to time constraints, only the VP1 region was analysed and studied. In future studies, we will conduct research on the complete genome sequence of EV71 in China. Second, the Chinese EV71VP1 strains registered in GenBank do not cover all provinces in the country, and the data for some years are missing; thus, some isolates of other genotypes may have been unavailable. Third, it is not clear whether some variations in the amino acid residues found in the study are related to the severity of disease or the route of transmission.

## Conclusion

In summary, the prevalent strains of EV71 belong mainly to the C4 genotype. The C4a subgenotype was predominant, the C4b subgenotype was the second most prevalent, whereas other subgenotypes appeared sporadically in mainland China. The B4 genotype was the dominant genotype in Taiwan, and the epidemic strain is continuously changing. Moreover, variation in key positions of the EV71VP1 region is very important for the development of severe HFMD. Taken together, the findings indicate that the genetic characteristics of the EV71VP1 region should be continuously monitored, which is essential for the prevention and control of EV71-associated HFMD in children and EV71 vaccine design.

## Data Availability

The available data used and/or analysed during the current study are all included in the manuscript.

## References

[1] Yang F, Ren L, Xiong Z (2009). Enterovirus 71 outbreak in the People’s Republic of China in 2008. J ClinMicrobiol..

[2] Wang Y, Feng Z, Yang Y (2011). Hand, foot, and mouth disease in China: patterns of spread and transmissibility. Epidemiology..

[3] Xing W, Liao Q, Viboud C (2014). Hand, foot, and mouth disease in China, 2008–12: an epidemiological study. Lancet Infect Dis..

[4] Solomon T, Lewthwaite P, Perera D (2010). Virology, epidemiology, pathogenesis, and control of enterovirus 71. Lancet Infect Dis..

[5] Luo Z, Su R, Wang W (2019). EV71 infection induces neurodegeneration via activating TLR7 signaling and IL-6 production. PLoSPathog..

[6] Antona D, Kossorotoff M, Schuffenecker I (2016). Severe paediatric conditions linked with EV-A71 and EV-D68, France, May to October 2016. Euro Surveill.

[7] Ooi MH, Wong SC, Lewthwaite P (2010). Clinical features, diagnosis, and management of enterovirus 71. Lancet Neurol..

[8] Kok CC (2015). Therapeutic and prevention strategies against human enterovirus 71 infection. World J Virol..

[9] Ho M, Chen ER, Hsu KH (1999). An epidemic of enterovirus 71 infection in Taiwan. Taiwan Enterovirus Epidemic Working Group. N Engl J Med.

[10] Shih SR, Ho MS, Lin KH (2000). Genetic analysis of enterovirus 71 isolated from fatal and non-fatal cases of hand, foot and mouth disease during an epidemic in Taiwan, 1998. Virus Res.

[11] Chan KP, Goh KT, Chong CY (2003). Epidemic hand, foot and mouth disease caused by human enterovirus 71, Singapore. Emerg Infect Dis..

[12] Herrero LJ, Lee CS, Hurrelbrink RJ (2003). Molecular epidemiology of enterovirus 71 in peninsular Malaysia, 1997–2000. Arch Virol.

[13] Guan D, van der Sanden S, Zeng H (2012). Population dynamics and genetic diversity of C4 strains of human enterovirus 71 in Mainland China, 1998–2010. PLoS ONE.

[14] Zhu Z, Zhu S, Guo X (2010). Retrospective seroepidemiology indicated that human enterovirus 71 and coxsackievirus A16 circulated wildly in central and southern China before large-scale outbreaks from 2008. Virol J..

[15] Wang JR, Tuan YC, Tsai HP (2002). Change of major genotype of enterovirus 71 in outbreaks of hand-foot-and-mouth disease in Taiwan between 1998 and 2000. J Clin Microbiol.

[16] Yu H, Chen W, Chang H (2010). Genetic analysis of the VP1 region of enterovirus 71 reveals the emergence of genotype A in central China in 2008. Virus Genes.

[17] van der Sanden S, van der Avoort H, Lemey P (2010). Evolutionary trajectory of the VP1 gene of human enterovirus 71 genogroup B and C viruses. J GenVirol..

[18] Zhang Y, Zhu Z, Yang W (2010). An emerging recombinant human enterovirus 71 responsible for the 2008 outbreak of hand foot and mouth disease in Fuyang city of China. Virol J..

[19] Lee KY (2016). Enterovirus 71 infection and neurological complications. Korean J Pediatr..

[20] Tan X, Huang X, Zhu S (2011). The persistent circulation of enterovirus 71 in People’s Republic of China: causing emerging nationwide epidemics since 2008. PLoS ONE.

[21] Tao Z, Wang H, Xu A (2012). Identification of a C2 subgenogroup strain of enterovirus 71 in a retrospective study in Shandong Province, China, from 1990 to 2010. J Clin Microbiol.

[22] Tao Z, Wang H, Li Y (2014). Molecular epidemiology of human enterovirus associated with aseptic meningitis in Shandong Province, China, 2006–2012. PLoS ONE.

[23] Yip CC, Lau SK, Woo PC (2013). Human enterovirus 71 epidemics: what’s next?. EmergHealth Threats J..

[24] Chia MY, Chiang PS, Chung WY (2014). Epidemiology of enterovirus 71 infections in Taiwan. PediatrNeonatol..

[25] Huang SW, Cheng HL, Hsieh HY (2014). Mutations in the non-structural protein region contribute to intra-genotypic evolution of enterovirus 71. J Biomed Sci.

[26] Chong P, Liu CC, Chow YH (2015). Review of enterovirus 71 vaccines. Clin Infect Dis.

[27] Chou AH, Liu CC, Chang JY (2013). Formalin-inactivated EV71 vaccine candidate induced cross-neutralizing antibody against subgenotypes B1, B4, B5 and C4A in adult volunteers. PLoS ONE.

[28] Bible JM, Iturriza-Gomara M, Megson B (2008). Molecular epidemiology of human enterovirus 71 in the United Kingdom from 1998 to 2006. J Clin Mocrobiol..

[29] Huang SC, Hsu YW, Wang HC (2008). Appearance of intratypic recombination of enterovirus 71 in Taiwan from 2002 to 2005. Virus Res.

[30] van der Sanden S, van Eek J, Martin DP (2011). Detection of recombination breakpoints in the genomes of human enterovirus 71 strains isolated in the Netherlands in epidemic and non-epidemic years, 1963–2010. Infect Genet Evol..

[31] McWilliam LE, Cabrerizo M, Cardosa J (2012). The association of recombination events in the founding and emergence of subgenogroup evolutionary lineages of human enterovirus 71. J Virol.

[32] Wen S, Ma D, Lin Y (2018). Complete genome characterization of the 2017 dengue outbreak in Xishuangbanna, a Border City of China, Burma and Laos. Front Cell Infect Microbiol..

[33] Zhang Y, Tan X, Cui A (2013). Complete genome analysis of the C4 subgenotype strains of enterovirus 71: predominant recombination C4 viruses persistently circulating in China for 14 years. PLoS ONE.

[34] Li Y, Zhou Y, Cheng Y (2019). Effectiveness of EV-A71 vaccination in prevention of paediatric hand, foot, and mouth disease associated with EV-A71 virus infection requiring hospitalisation in Henan, China, 2017–18: a test-negative case-control study. Lancet Child Adolesc Health..

[35] Weng Y, Chen W, Huang M (2017). Epidemiology and etiology of hand, foot, and mouth disease in Fujian province, 2008–2014. Arch Virol.

[36] Liu Y, Fu C, Wu S (2014). A novel finding for enterovirus virulence from the capsid protein VP1 of EV71 circulating in mainland China. Virus Genes.

[37] Wu JS, Zhao N, Pan H (2013). Patterns of polymorphism and divergence in the VP1 gene of enterovirus 71 circulating in the Asia-Pacific region between 1994 and 2013. J Virol Methods.

[38] Zhu H, Cao Y, Su W (2019). Enterovirus A71 VP1 variation A289T decreases the central nervous system infectivity via attenuation of interactions between VP1 and vimentin in vitro and in vivo. Viruses..

[39] Nishimura Y, Lee H, Hafenstein S (2013). Enterovirus 71 binding to PSGL-1 on leukocytes: VP1-145 acts as a molecular switch to control receptor interaction. PLoS Pathog..

[40] Chen TC, Liu SC, Huang PN (2008). Antiviral activity of pyridyl imidazolidinones against enterovirus 71 variants. J Biome Sci..

[41] Le TV, Nguyen V, Nguyen QH (2019). Molecular epidemiology analysis of enterovirus 71 strains isolated in Dak Lak, Vietnam, 2011–2016. J Med Virol.

[42] Zhang B, Wu X, Huang K (2014). The variations of VP1 protein might be associated with nervous system symptoms caused by enterovirus 71 infection. BMC Infect Dis.

[43] Kobayashi K, Sudaka Y, Takashino A (2018). Amino acid variation at VP1-145 of Enterovirus 71 determines attachment receptor usage and neurovirulence in human scavenger receptor B2 transgenic mice. J Virol.

[44] Victorio CB, Xu Y, Ng Q (2016). Cooperative effect of the VP1 amino acids 98E, 145A and 169F in the productive infection of mouse cell lines by enterovirus 71 (BS strain). Emerg Microbes Infect..

[45] Yang CH, Liang CT, Jiang ST (2019). A novel murine model expressing a chimeric mSCARB2/hSCARB2 receptor is highly susceptible to oral infection with clinical isolates of Enterovirus 71. J Virol.

[46] Zhang H, Song Z, Zou J (2020). An infectious clone of enterovirus 71(EV71) that is capable of infecting neonatal immune competent mice without adaptive mutations. Emerg Microbes Infect..

[47] Zhu FC, Meng FY, Li JX (2013). Efficacy, safety, and immunology of an inactivated alum-adjuvant enterovirus 71 vaccine in children in China: a multicentre, randomised, double-blind, placebo-controlled, phase 3 trial. Lancet.

[48] Mao QY, Wang Y, Bian L (2016). EV71 vaccine, a new tool to control outbreaks of hand, foot and mouth disease (HFMD). Expert Rev Vaccines..

[49] Yang B, Liu F, Liao Q (2017). Epidemiology of hand, foot and mouth disease in China, 2008 to 2015 prior to theintroduction of EV-A71 vaccine. Euro Surveill.

[50] Li R, Liu L, Mo Z (2014). An inactivated enterovirus 71 vaccine in healthy children. N Engl J Med.

[51] Zhu F, Xu W, Xia J (2014). Efficacy, safety, and immunogenicity of an enterovirus 71 vaccine in China. N Engl J Med.

[52] Hu YM, Wang X, Wang JZ (2013). Immunogenicity, safety, and lot consistency of a novel inactivated enterovirus 71 vaccine in Chinese children aged 6 to 59 months. Clin Vaccine Immunol.

[53] Bessaud M, Razafindratsimandresy R, Nougairede A (2014). Molecular comparison and evolutionary analyses of VP1 nucleotide sequences of new African human enterovirus 71 isolates reveal a wide genetic diversity. PLoS ONE.

